# The inclusion and selection of underserved participants for interventional microbiota trials involving cognitively impaired older adults

**DOI:** 10.1002/alz70860_100453

**Published:** 2025-12-23

**Authors:** Cynthia Williams, Adam Golden, Hariom Yadav, Michal M Masternak, Microbiome in Aging Gut Consortium

**Affiliations:** ^1^ University of Central Florida, ORLANDO, FL, USA; ^2^ University of Central Florida, Orlando, FL, USA; ^3^ Center for Microbiome Research, Microbiomes Institute, University of South Florida, Tampa, FL, USA

## Abstract

**Background:**

Gut microbiota is crucial in nutrient extraction, metabolism, cognition, and immune function. Consequently, the increasing number of microbiome studies aims to link specific bacteria, fungi, and viruses with various cognitive disease outcomes. Unfortunately, clinical studies often exclude many older adults with Alzheimer's Disease and Related Dementia who are homebound or from racially/ethnically diverse populations. The homebound older adult population is estimated to be three times larger than the equally impaired and chronically ill nursing home population. People of color often hesitate to participate in clinical trials due to mistrust, logistical barriers, and lack of awareness. Recruiting a diverse group of patients has been challenging.

**Method:**

We review the literature using CINAL, PubMed, Medline, PsycINFO, and Embase to highlight evidence‐based strategies for promoting inclusivity among homebound and minority communities in microbiome studies. Additionally, we discussed the inclusion and exclusion criteria necessary for clinical trials.

**Results:**

We identified strategies such as community engagement, culturally appropriate assistance, mobile health units, and strategic partnerships with feedback mechanisms to improve recruitment and retention of underserved populations. We also discussed inclusion and exclusion criteria while highlighting factors that can confound results. While these criteria may complicate trials involving vulnerable populations, they are essential for optimizing outcomes. We must recognize and adequately support these populations while keeping these criteria in mind.

**Conclusion:**

This review emphasized recruitment strategies for underrepresented groups in microbiome studies and underscores the importance of inclusion and exclusion criteria to ensure robust study results. Without inclusivity in microbiota clinical trials, we cannot effectively address health inequities or ensure the generalizability of findings. The complexity and long‐term nature of these trials suggest that additional support for patients and caregivers may be necessary for participants with cognitive decline. Diverse participation helps uncover variations in disease prevalence, progression, and treatment responses among different populations, leading to more personalized and effective healthcare solutions. It also enhances the overall quality of research by incorporating a wide range of perspectives and experiences.

